# Supplementary material for: Gold nanoparticles enhance antibody effect through direct cancer cell cytotoxicity by differential regulation of phagocytosis
Source: Nat Commun. 2021 Nov 4;12:6371. doi: 10.1038/s41467-021-26694-x (PMC8569206; doi:10.1038/s41467-021-26694-x)
Supplement: Supplementary file 1 — Supplementary Information [file 41467_2021_26694_MOESM1_ESM.docx]

**Supporting Information**

**Gold nanoparticles enhanced antibody effect through direct cancer cell cytotoxicity by differential regulation of phagocytosis**

***Linyang Fan#^1, 2^and Weizhi Wang^2^, Zihua Wang****^3^****, Minzhi Zhao*^1^***

1. CAS Key Laboratory for Biomedical Effects of Nanomaterials and Nanosafety, CAS Center for Excellence in Nanoscience, National Center for Nanoscience and Technology of China, Beijing 100190, China
2. School of Chemistry and Chemical Engineering, Beijing Institute of Technology, Beijing 100081, China
3. Centre for Neuroscience Research, School of Basic Medical Sciences, Fujian Medical University, Fuzhou 350108, Fujian, PR China;

*****Correspondence should be addressed to Minzhi Zhao ([xgt1986627@163.com](mailto:xgt1986627@163.com))

**Ramucirumab labeled Cy5 purification by HPLC.**

Ramucirumab was labeled by Cy5 using NHS water labeling of an amino biomolecules kit. briefly, 100 μL 20 mg/mL antibody added 800 μL 0.1M NaHCO_3_ in microcentrifuge tube, dissolve NHS ester (1 μg/mL) 66 μL into tube and vortex well, incubate the mixture overnight at room temperature. Purify the conjugate using gel-filtration method. SEC-HPLC analyze the product and check the sharp and retention time of prominent peak. **Supplementary Figure1** showed that retention time of Ab-Cy5 had been moved up (Molecular Weight was increased ) and shoulder peak appeared(Ab and Ab-Cy5 mixture).





**Supplementary Figure 1** SEC-HPLC analysis of Antibody and antibody labeled by Cy5

**Ellman method analyze.**

Standard calibration curve for PEG chains, whose concentration can be calculated via the following equation: Abs at 412nm = 0.0895×[PEG, mg/mL]+0.4967, R^2^=0.999. Variation of the excess of PEG thiolate chains as a function of the initial concentration in the incubation with 1 mL of nanorods. The dashed vertical line indicates the 100% saturation, i.e. the PEG concentration above which no more PEG can be bound to the nanoparticle’s surface.





**Supplementary Figure 2** Ellman’s Assay evaluate functionalization of gold nanorods with poly (ethylene glycol) (Peg)


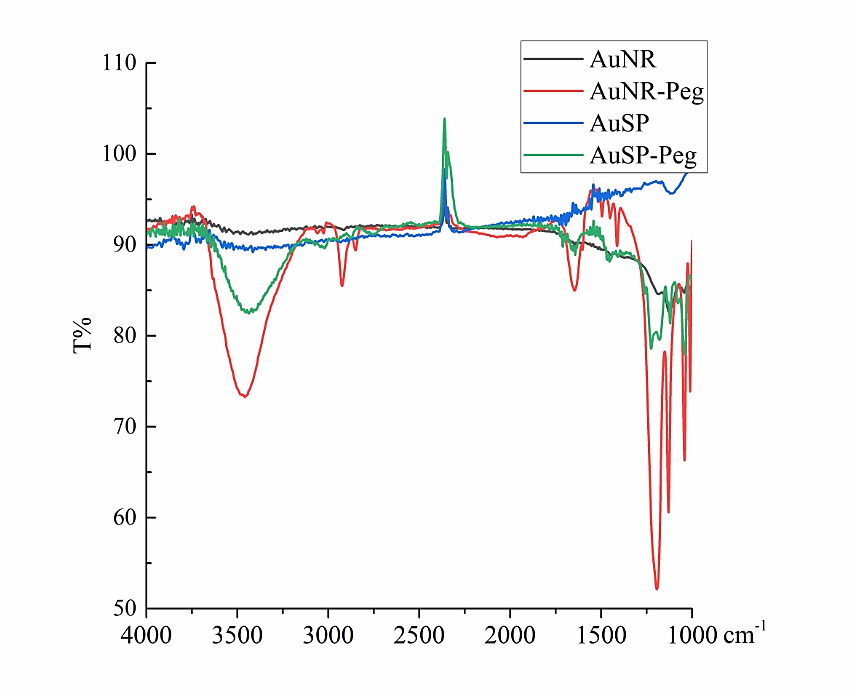


**Supplementary Figure 3 FTIR spectra analysis of Au nanoparticles and Au-PEG.**

**Selection for differential protein and expressed genes in the proteomics and transcriptomics.**

The analyses were based on diﬀerentially proteins and expressed genes in each treatment groups. Diﬀerential expression was defined as multiple testing adjusted p values smaller than or equal to 0.05 and fold change greater than or equal to 2.0-fold.


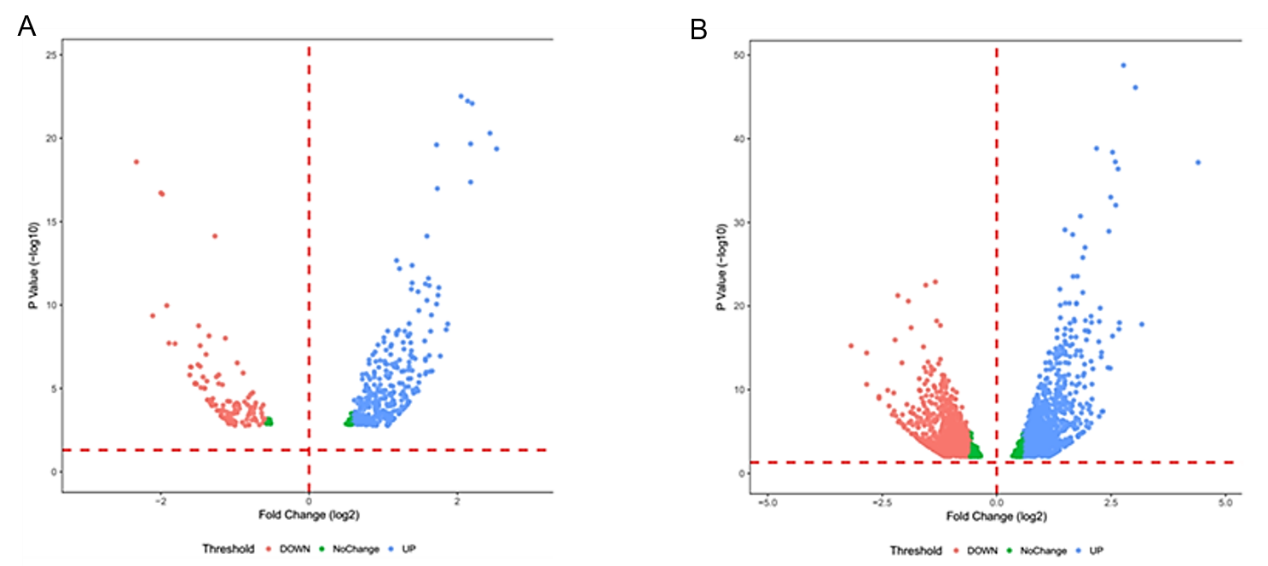


**Supplementary Figure 4.** Volcano plots of Quantitative proteomics (A) and transcriptomics (B) results. The volcano plots were assembled which represented that the highly deregulated proteins and genes marked with red (SP group) or blue (NR group) color appear in the left or right sides. They showed the different expression genes for the different groups.


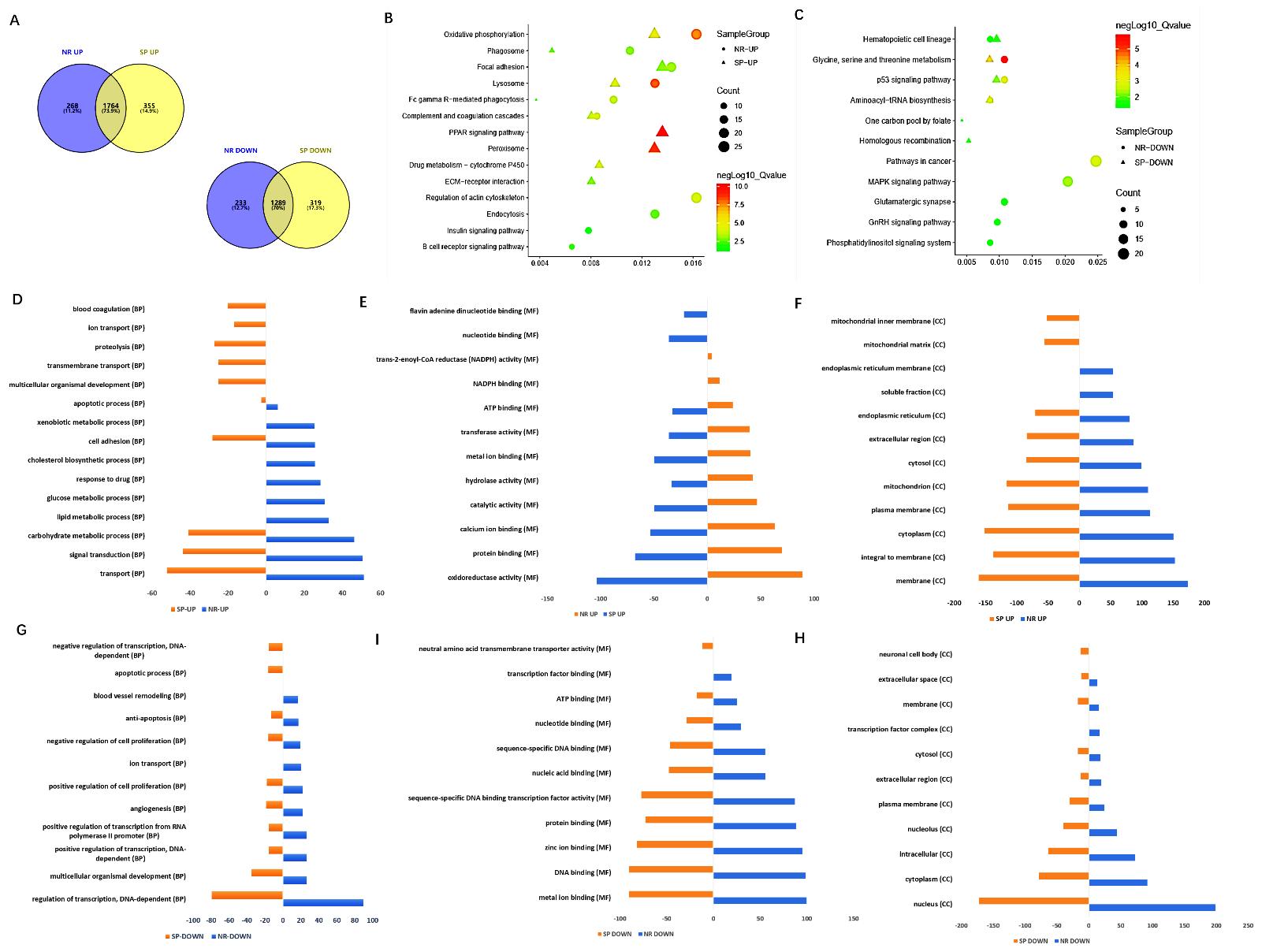


**Supplementary Figure 5.** Transcriptomics analysis of AuNR-Peg-Ab (NR group) and AuSP-Peg-Ab (SP group) treated groups compared with Ab treated group respectively in SNU5 cell. A) Venn grams of the whole numbers of differential expressed genes quantified in transcriptomics results from different comparations. B-C) KEGG enrichment of differential genes. the color indicated the level of q value and the items with q<0.05 were included. D-F) Top 10 items in the Gene Ontology (GO) biological process (BP), molecular function (MF), cellular component (CC) enrichment of up-regulated genes in the different comparisons. G-H) Top 10 items in the Gene Ontology (GO) biological process (BP), molecular function (MF), cellular component (CC) enrichment of down-regulated genes in the different comparisons.

**
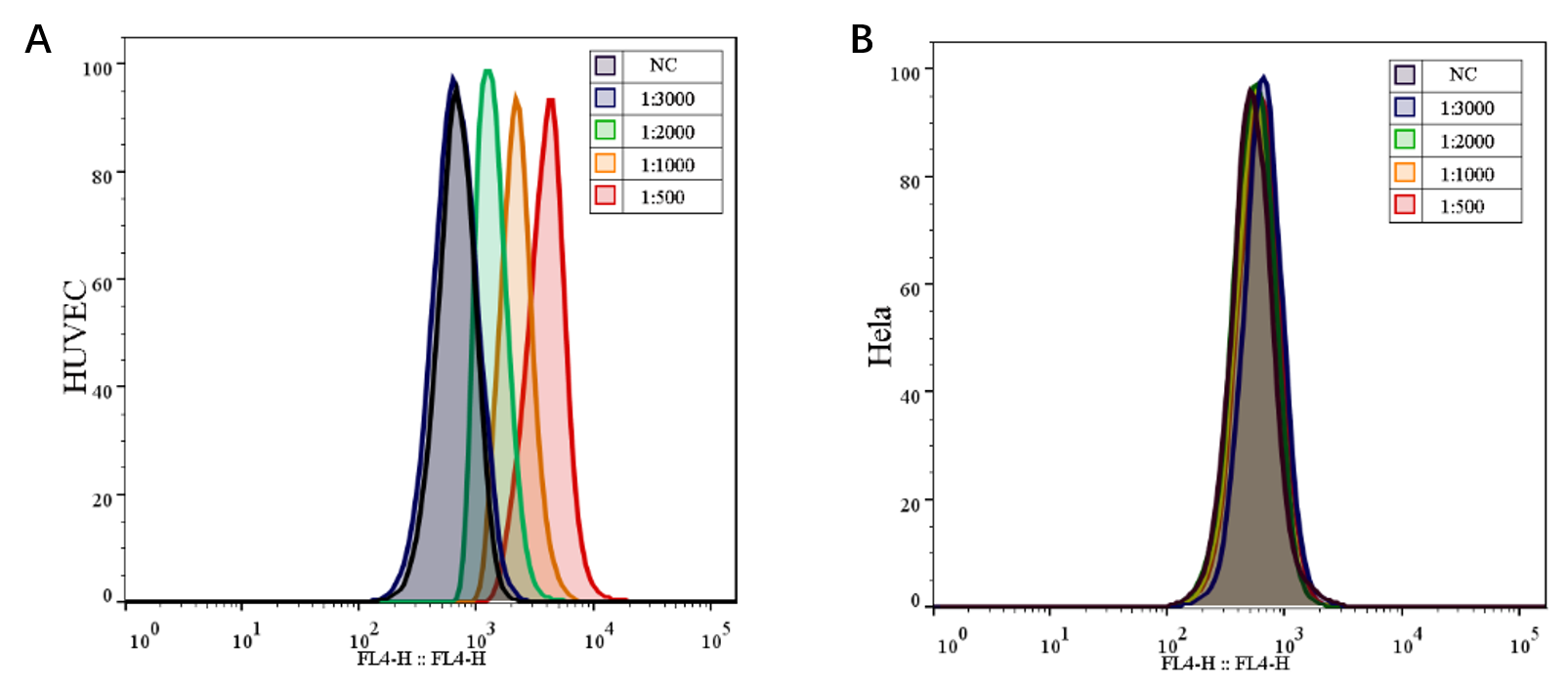
**

**Supplementary Figure 6.** A-B), Flow cytometry analysis of Ab in HUVEC and Hela cells.

**
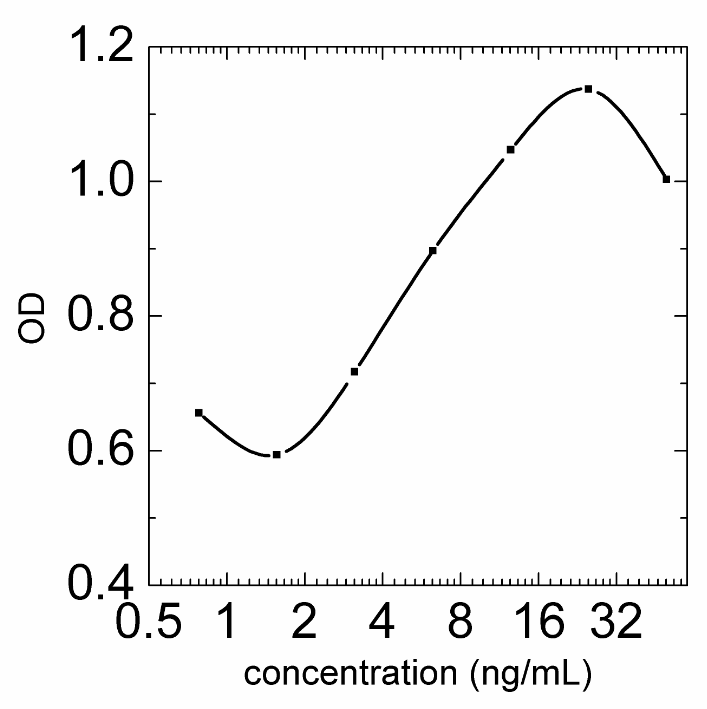
**

**Supplementary Figure 7.** Cell viability analysis on VEGF treated HUVEC cells

**
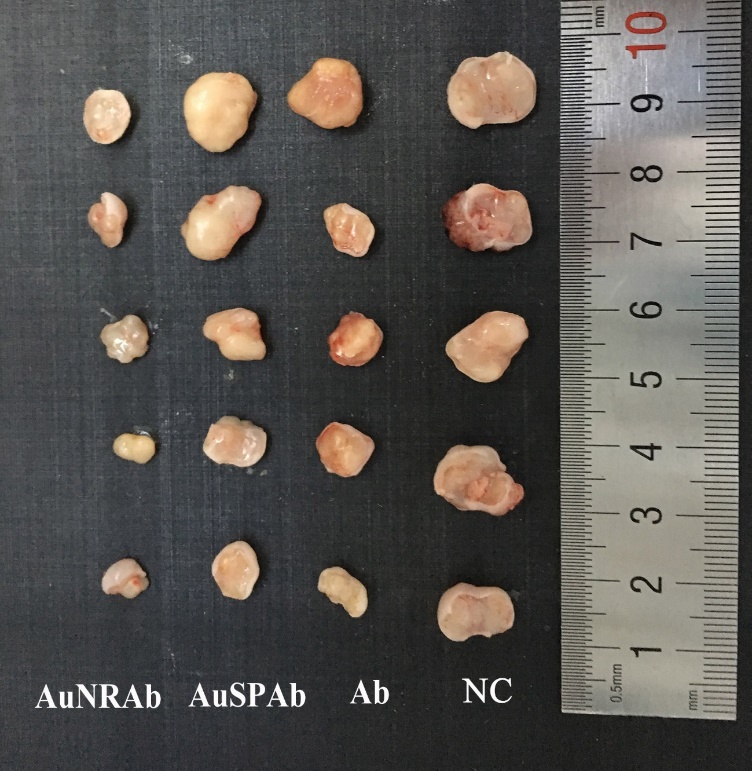
**

**Supplementary Figure 8 Original picture of mice tumors from different treated groups**


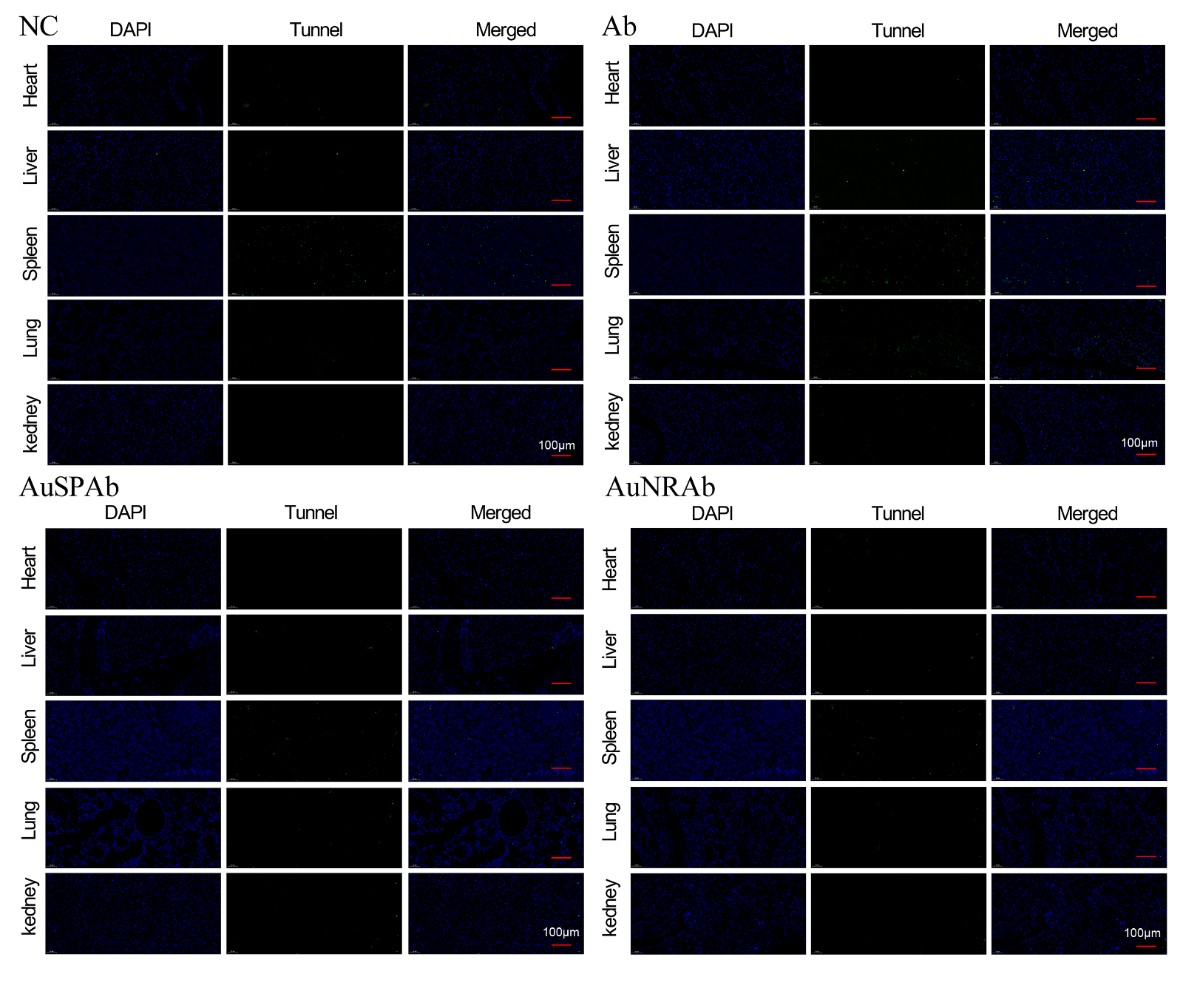


**Supplementary Figure 9** Representative Tunnel immunofluorescence straining images to several tissues


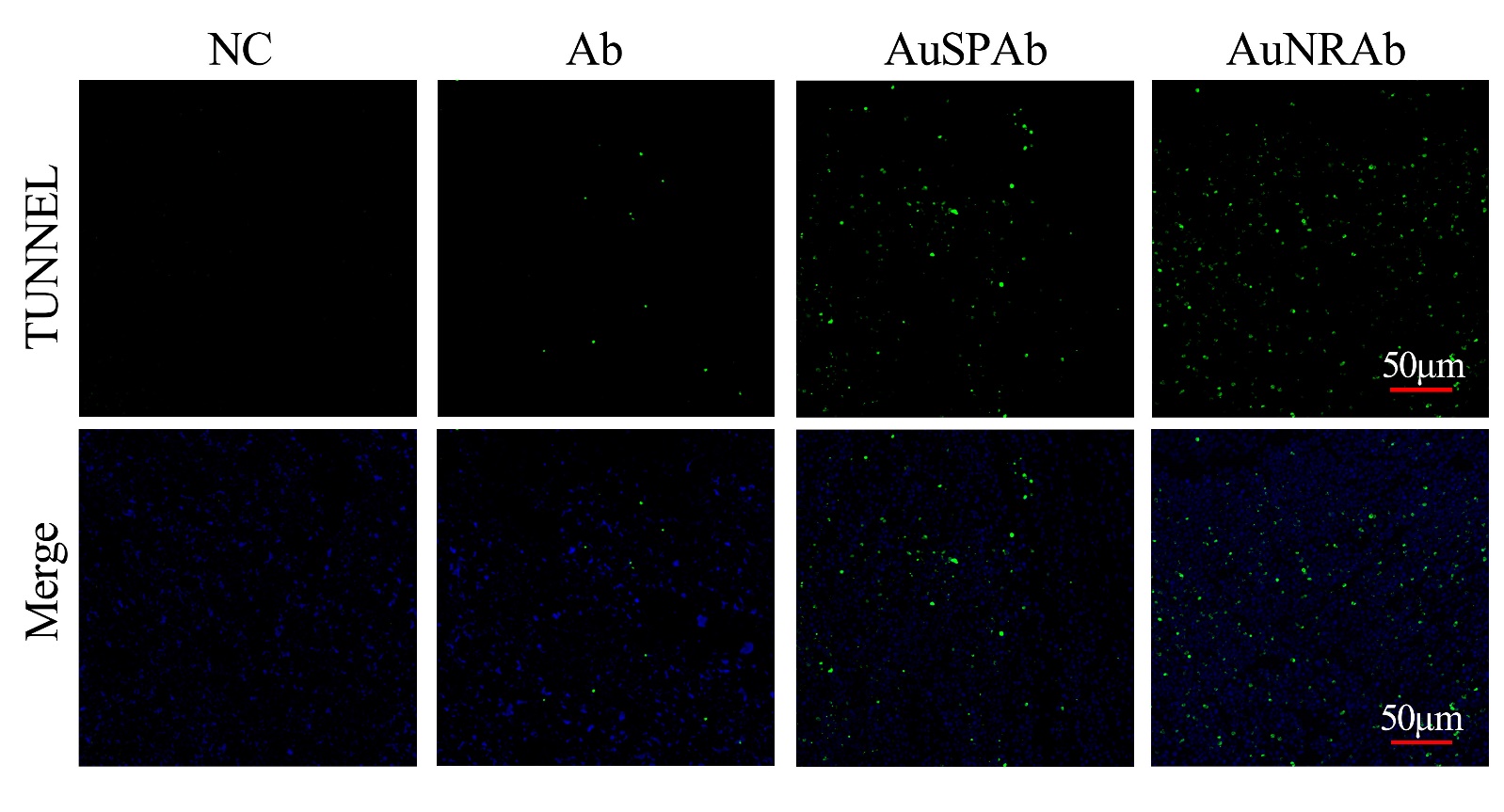


**Supplementary Figure 10** Representative Tunnel immunofluorescence straining to tumor (**Figure 6D**)


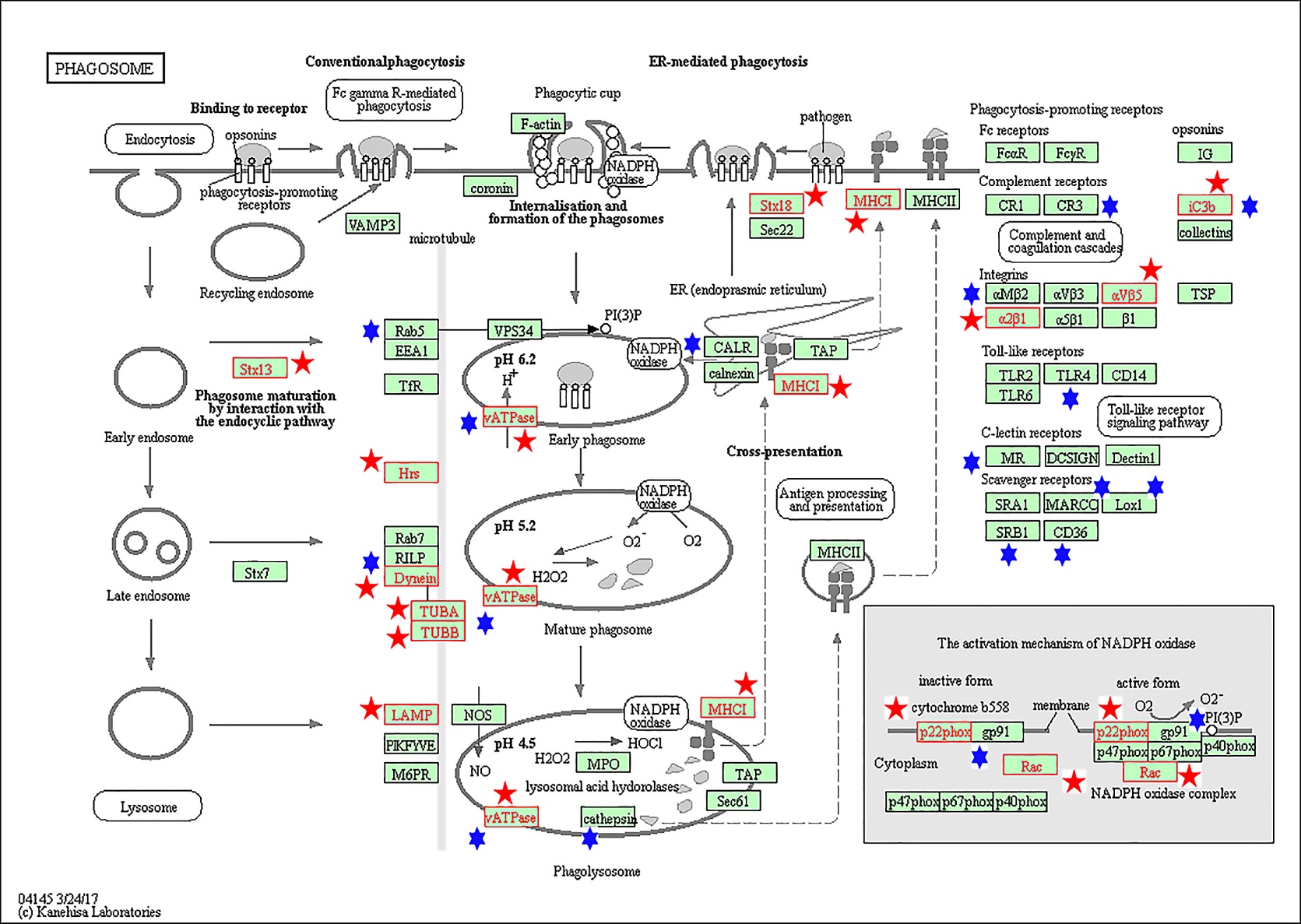


**Supplementary Figure 11** Pathway “phagosome” from KEGG with altered proteins (red pentagram) and expressed genes (blue hexagram) mapped in it (**Figure 6D**)

**
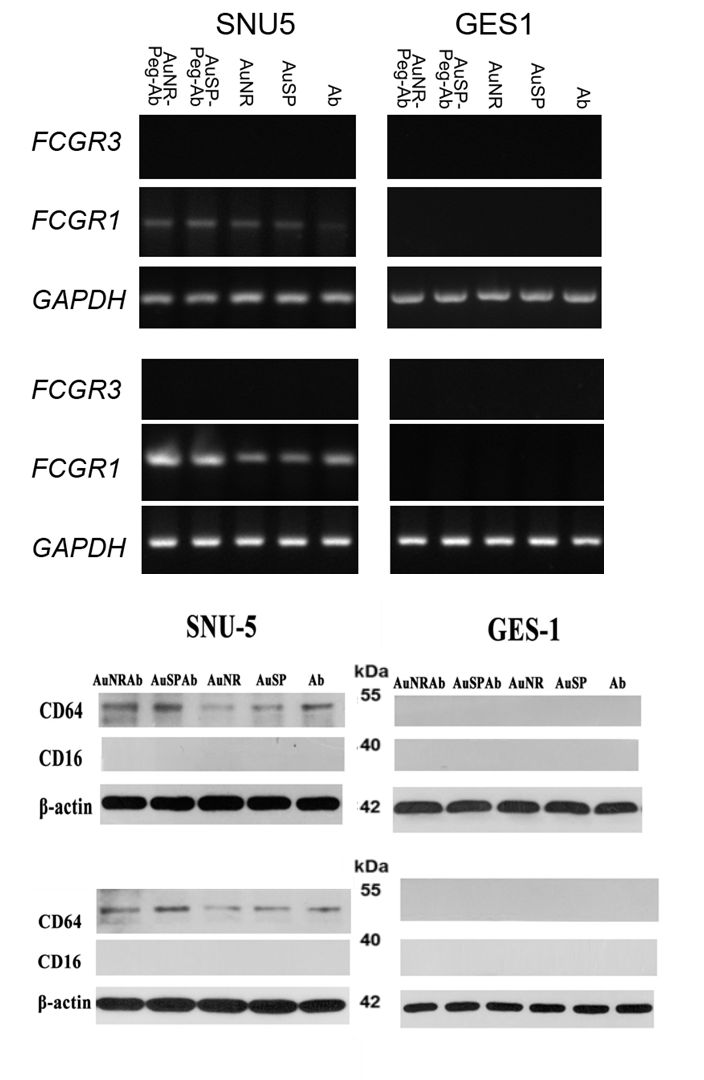
**

**Supplementary Figure 12** All the repeated RT-PCR (up) and western-blot results (down). Three times were repeated independently.


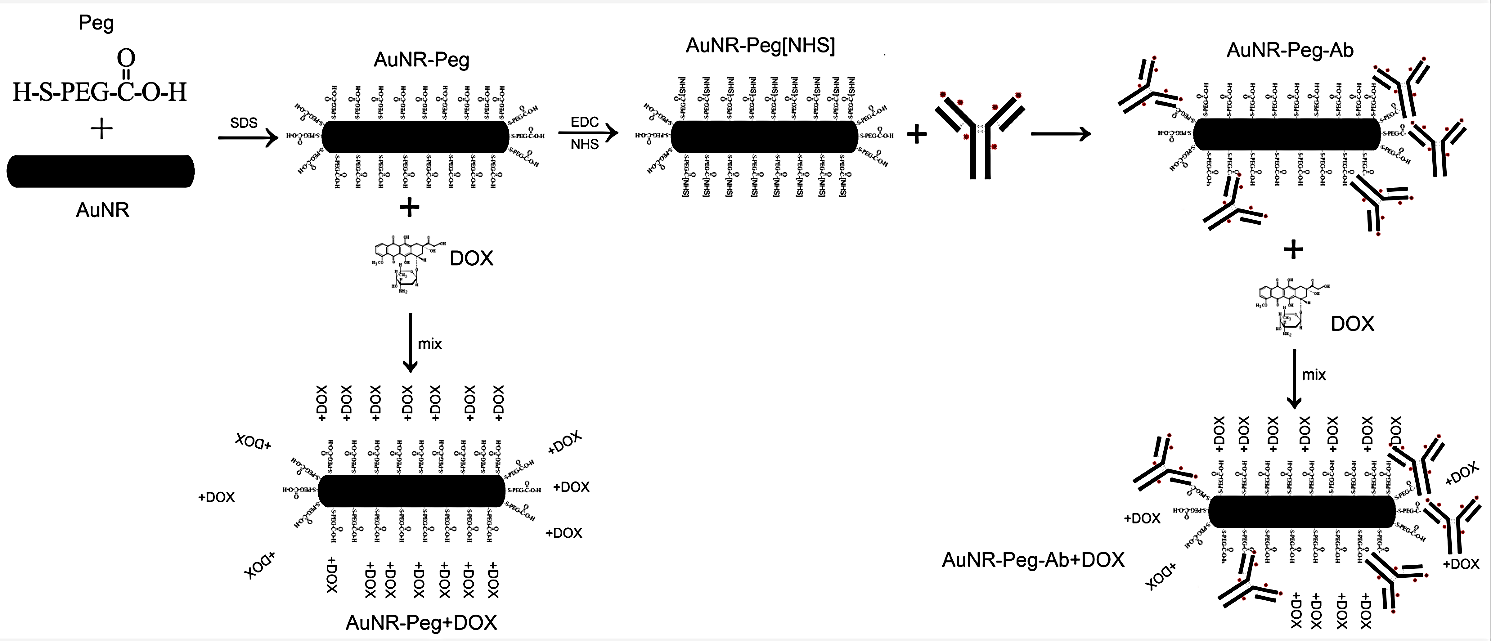


**Supplementary Figure13** Schematic diagram of material synthesis process (All drawings are not in scale)

**
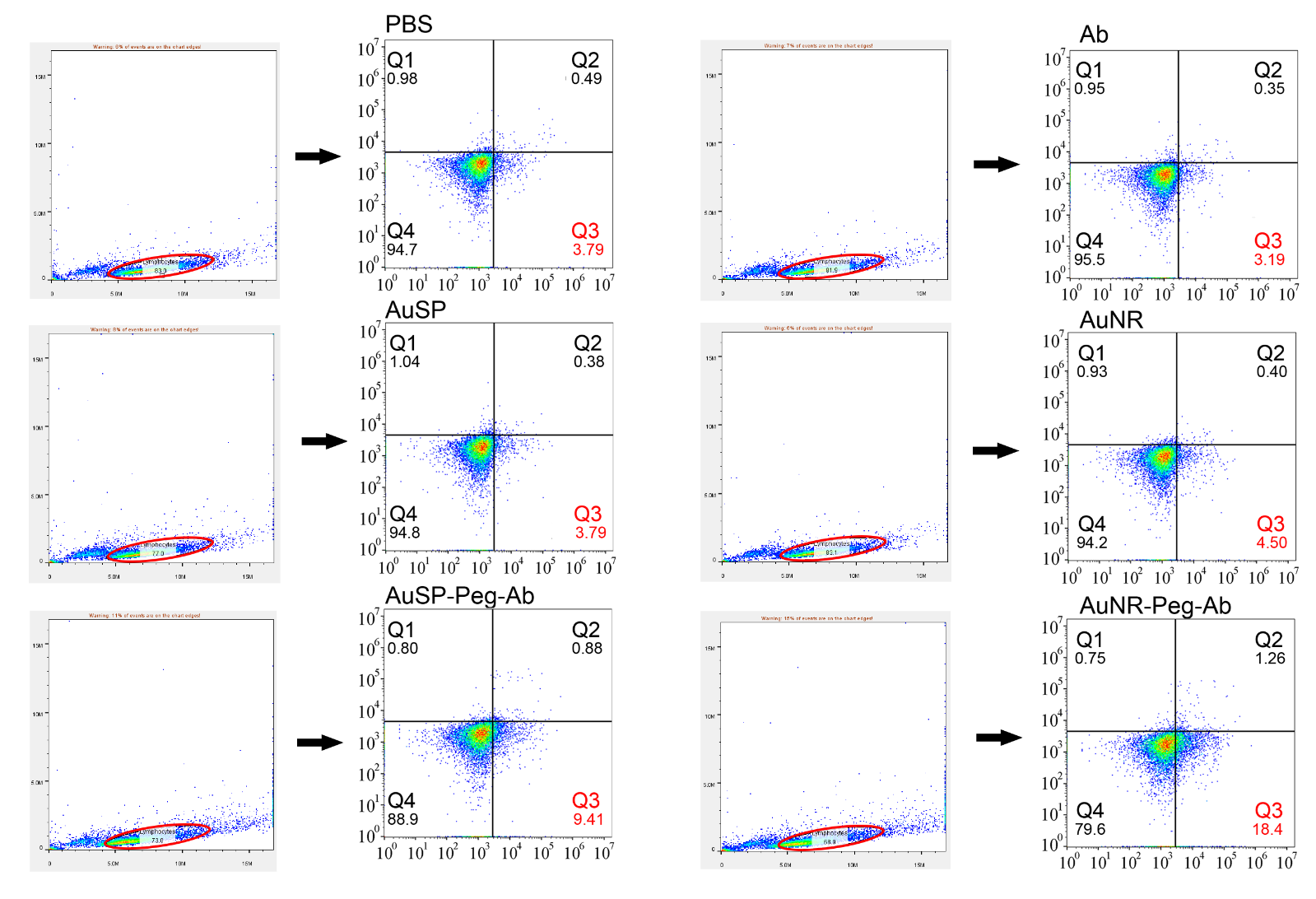
**

**Supplementary Figure14** The gate setting schematic for cell apoptosis analysis by flow cytometry of SNU5 cells in Figure 3M.

**
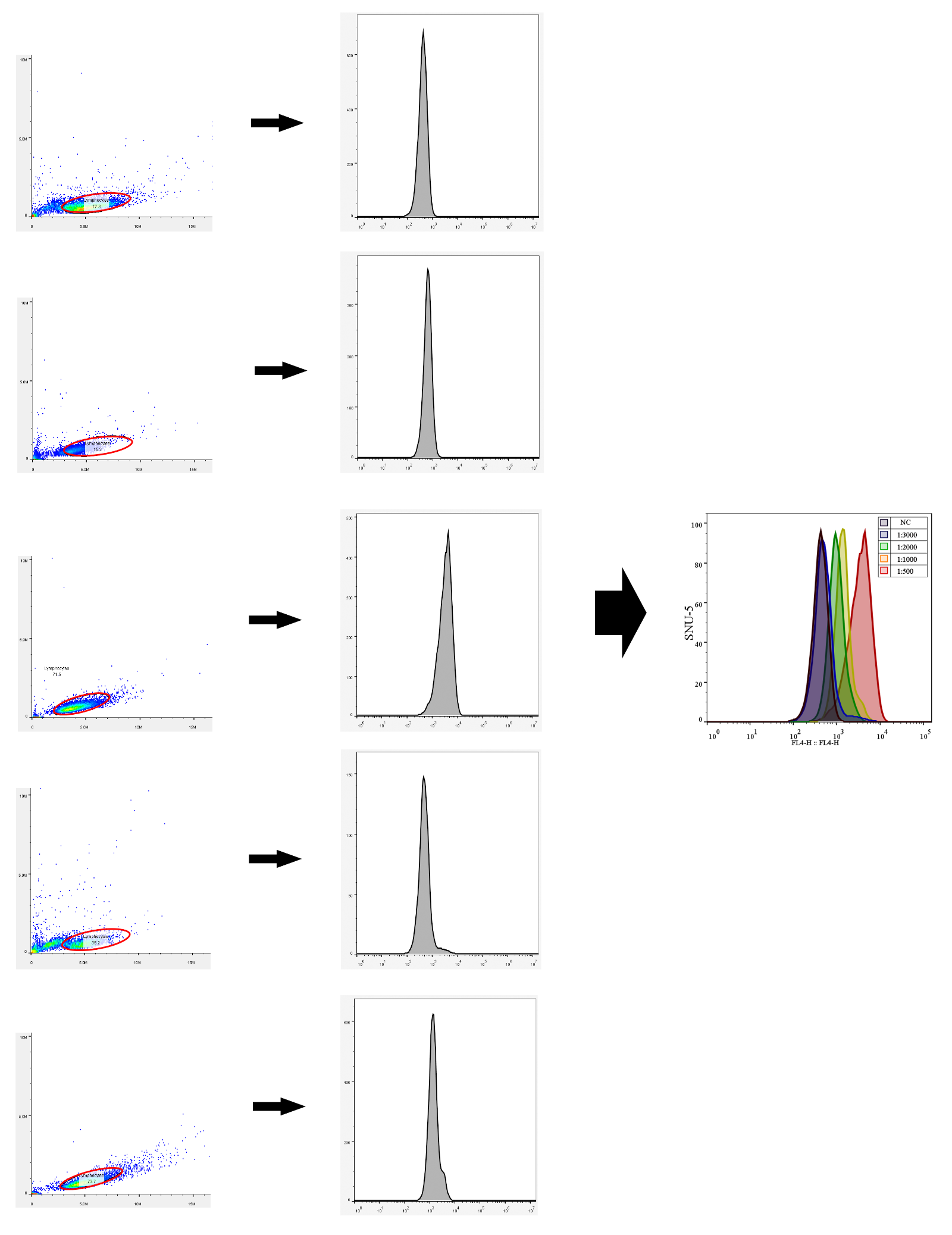
**

**Supplementary Figure15** The gate setting schematic for vegfr2 expression by flow cytometry of SNU5 cells in Figure 5F.

**
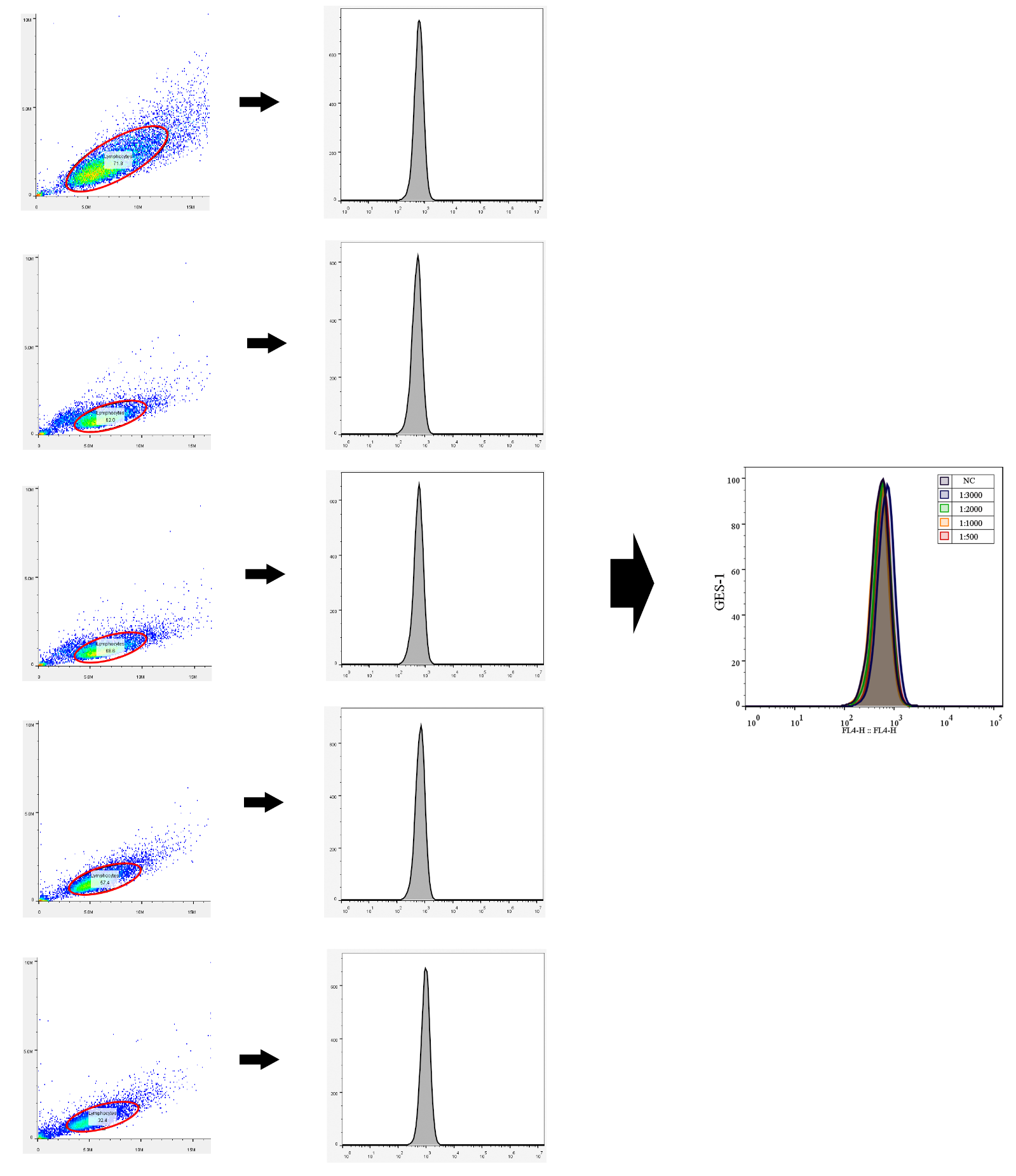
**

**Supplementary Figure16** The gate setting schematic for vegfr2 expression by flow cytometry of GES-1 cells in Figure 5F.

**Supplementary Table 1** Physical-chemical properties of functionalized gold nanoparticles

| Formulation | hydrodynamic diameters (nm) | Zeta-Potential (mV) | mol Ab per  particle |
| --- | --- | --- | --- |
| AuSP | 25.2±1.9 | -9.06±1.2 | NA |
| AuNR | 28.6±2.7 | -6.7±2.1 | NA |
| AuSP-PEG | 31.5±3.3 | -20.88±2.8 | NA |
| AuNR-PEG | 36.2±3.9 | -26.1±3.9 | NA |
| AuSP-PEG-Ab | 37.8±4.3 | -21.7±3.0 | 1 μg/mL |
| AuNR-PEG-Ab | 39.3±5.1 | -29.2±3.4 | 1 μg/mL |

**Supplementary Table 2 All primers used in PT-PCR**

| name | seq | product length |
| --- | --- | --- |
| FCGR1-F | TAAGTCACAATGGCACCTACC | 306 |
| FCGR1-R | CCTCGCACCAGTATAACCC |  |
| FCGR3-F | GGAGAAAGGACCCTCAAGAC | 334 |
| FCGR3-R | TACCATCCCTAGCCTGTATTGT |  |
| GAPDH-F | GAATGGGCAGCCGTTAG | 354 |
| GAPDH-R | TGGAAGATGGTGATGGGAT |  |
