# Supplementary material for: Gold nanoparticles enhance antibody effect through direct cancer cell cytotoxicity by differential regulation of phagocytosis
Source: Nat Commun. 2021 Nov 4;12:6371. doi: 10.1038/s41467-021-26694-x (PMC8569206; doi:10.1038/s41467-021-26694-x)
Supplement: Supplementary file 3 — Description of Additional Supplementary Files [file 41467_2021_26694_MOESM3_ESM.docx]

**Description of Additional Supplementary Files**

**Supplementary Data 1:** Summary of proteomic analysis results NR group (AuNR-Peg-Ab) and SP group (AuSP-Peg-Ab).

**Supplementary Data 2:** Summary of transcriptomic analysis results NR group (AuNR-Peg-Ab), SP group (AuSP-Peg-Ab) and Ramucirumab (Ab) treated group.
