# Supplementary material for: Gold nanoparticles enhance antibody effect through direct cancer cell cytotoxicity by differential regulation of phagocytosis
Source: Nat Commun. 2021 Nov 4;12:6371. doi: 10.1038/s41467-021-26694-x (PMC8569206; doi:10.1038/s41467-021-26694-x)
Supplement: Supplementary file 6 — Reporting Summary [file 41467_2021_26694_MOESM6_ESM.pdf]

nature portfolio

Corresponding author(s): Minzhi Zhao

Last updated by author(s): Jul 20, 2021

Reporting Summary

Nature Portfolio wishes to improve the reproducibility of the work that we publish. This form provides structure for consistency and transparency in reporting. For further information on Nature Portfolio policies, see our Editorial Policies and the Editorial Policy Checklist.

Statistics

For all statistical analyses, confirm that the following items are present in the figure legend, table legend, main text, or Methods section.

n/a	Confirmed	
☐	☒	The exact sample size (n) for each experimental group/condition, given as a discrete number and unit of measurement
☐	☒	A statement on whether measurements were taken from distinct samples or whether the same sample was measured repeatedly
☐	☒	The statistical test(s) used AND whether they are one- or two-sided<br>*Only common tests should be described solely by name; describe more complex techniques in the Methods section.*
☒	☐	A description of all covariates tested
☐	☒	A description of any assumptions or corrections, such as tests of normality and adjustment for multiple comparisons
☐	☒	A full description of the statistical parameters including central tendency (e.g. means) or other basic estimates (e.g. regression coefficient) AND variation (e.g. standard deviation) or associated estimates of uncertainty (e.g. confidence intervals)
☐	☒	For null hypothesis testing, the test statistic (e.g. F, t, r) with confidence intervals, effect sizes, degrees of freedom and P value noted<br>*Give P values as exact values whenever suitable.*
☒	☐	For Bayesian analysis, information on the choice of priors and Markov chain Monte Carlo settings
☒	☐	For hierarchical and complex designs, identification of the appropriate level for tests and full reporting of outcomes
☒	☐	Estimates of effect sizes (e.g. Cohen's d, Pearson's r), indicating how they were calculated

Our web collection on statistics for biologists contains articles on many of the points above.

Software and code

Policy information about availability of computer code

Data collection	OriginPro2018, SPSS 19.0 and ImageJ 1.48v, FlowJo X 10.0.7r2
Data analysis	High-throught RNA sequencing read quality was evaluated by FastQC (v0.11.4) and then they mapped to the human reference genome sequence (hg19, Genome Reference Consortium GRCh37) using Hisat2 , samtools and HTseq with Cufflinks software calculating FPKM. Proteomics data produced were searched using MaxQuant software (Computational Systems Biochemistry, Martinsried, Germany) package (version 1.5.1.2), against the SwissProt_2016_04 database with taxonomy of [human] selected. Heat maps were produced using Perseus (1.6.2.2). Gene ontology and KEGG pathway analyses were performed using GeneCodis 3.0.

For manuscripts utilizing custom algorithms or software that are central to the research but not yet described in published literature, software must be made available to editors and reviewers. We strongly encourage code deposition in a community repository (e.g. GitHub). See the Nature Portfolio guidelines for submitting code & software for further information.

Data

Policy information about availability of data

All manuscripts must include a data availability statement. This statement should provide the following information, where applicable:

- Accession codes, unique identifiers, or web links for publicly available datasets
- A description of any restrictions on data availability
- For clinical datasets or third party data, please ensure that the statement adheres to our policy

The data that support the findings of this study are available within the paper and its supplementary information files. The source data underlying Figure 2c-d, 3g-h, 4a-h, i-l, 6a-e, 7a-b, 7d and Supplementary Figs 1, 3 and 12 are provided as a Source Data file. The data that support he findings of this study are available from the corresponding author on request. Source data are provided with this paper. The proteomic raw data generated in this study have been deposited in the

ProteomeXchange database under accession code PXD027484. [http://proteomecentral.proteomexchange.org/cgi/GetDataset?ID=PXD027484]. The transcriptomic raw data generated in this study have been deposited in the GEO database under accession code GSE181369. [https://www.ncbi.nlm.nih.gov/geo/query/acc.cgi?acc=GSE181369].

Field-specific reporting

Please select the one below that is the best fit for your research. If you are not sure, read the appropriate sections before making your selection.

- [x] Life sciences
- [ ] Behavioural & social sciences
- [ ] Ecological, evolutionary & environmental sciences

For a reference copy of the document with all sections, see nature.com/documents/nr-reporting-summary-flat.pdf

Life sciences study design

All studies must disclose on these points even when the disclosure is negative.

Sample size	The sample size (n) of each experiment is provided in the corresponding figure captions in the main manuscript and supplementary information files. Sample size choice was based on previous studies (Ref 26), not predetermined by the statistical method.
Data exclusions	No animals and/or data were excluded.
Replication	All in vitro experiments were replicated independently for at least 3 times. In vivo sample size (n) in each group is detailed in the figure legends or methods section. All attempts at replication were successful.
Randomization	The different groups were filled by randomly selecting from the same pool of animals for in vivo experiments.
Blinding	All data collection and all related analyses were not performed blind. We followed standard laboratory procedures of randomization. Each experiment was designed with proper controls, and samples for comparison were collected and analyzed under the same conditions.

Reporting for specific materials, systems and methods

We require information from authors about some types of materials, experimental systems and methods used in many studies. Here, indicate whether each material, system or method listed is relevant to your study. If you are not sure if a list item applies to your research, read the appropriate section before selecting a response.

Materials & experimental systems

n/a	Involved in the study	
[ ]	[x]	Antibodies
[ ]	[x]	Eukaryotic cell lines
[x]	[ ]	Palaeontology and archaeology
[ ]	[x]	Animals and other organisms
[x]	[ ]	Human research participants
[x]	[ ]	Clinical data
[x]	[ ]	Dual use research of concern

Methods

n/a	Involved in the study	
[x]	[ ]	ChIP-seq
[ ]	[x]	Flow cytometry
[x]	[ ]	MRI-based neuroimaging

Antibodies

Antibodies used	Anti-CD64(EPR4623),abcam,1:1000 for WB;<br>Anti-CD16(EPR22409-124),abcam,1:1000 for WB;<br>Anti-beta Actin(mAbcam 8226),abcam,1:2000 for WB;<br>Anti-VEGFR2, Lilly, NDC 0002-7678-01, 1:500, 1:1000, 1:2000, 1:3000 for immunofluorescence staining
Validation	all antibody were purchant from commercal antibdoy and they have QC report from official website. anti-VEGFR2 was tested in HUVEC, GES-1, MKN-45 and SNU-5 cell lines.

Eukaryotic cell lines

Policy information about cell lines

Cell line source(s)	SNU-5, GES-1, HUVEC and MKN-45 were from the American Type Culture Collection (Manassas, VA, USA).
Authentication	Authentication of all Cells by the vendors was confirmed prior to their purchase. They were authenticated with STR profiling

Mycoplasma contamination	all cell lines have been tested negative for mycoplasma contamination by manufacturer.
Commonly misidentified lines (See ICLAC register)	No commonly misidentified cell lines were used.

Animals and other organisms

Policy information about studies involving animals; ARRIVE guidelines recommended for reporting animal research

Laboratory animals	mouse, Bal/bc female nude mice, 6-8week; female C57BL/6 mice, 6-8-week.
Wild animals	the study did not involve wild animals.
Field-collected samples	the study did not involve samples collected from the field
Ethics oversight	Animal Study Committee of the National Center for Nanoscience and Technology

Note that full information on the approval of the study protocol must also be provided in the manuscript.

Flow Cytometry

Plots

Confirm that:

- [x] The axis labels state the marker and fluorochrome used (e.g. CD4-FITC).
- [x] The axis scales are clearly visible. Include numbers along axes only for bottom left plot of group (a 'group' is an analysis of identical markers).
- [x] All plots are contour plots with outliers or pseudocolor plots.
- [x] A numerical value for number of cells or percentage (with statistics) is provided.

Methodology

Sample preparation	Cy5-labeled Ab (1mg/mL) was diluted by PBS(1:500, 1:1000, 1:2000, 1:3000). HUVEC and SNU5 cells incubated in Ab for 1h at RT. While SNU5 cells incubated in Ab, Peg-Ab, AuSP-Peg-Ab and AuNR-Peg-Ab(1μg/mL Ab) for 1h at RT. Then these cells were washed with PBS and re-suspended in PBS for analysis.
Instrument	C6 accuri, BD
Software	CFlowPlus and FlowJo software
Cell population abundance	10000
Gating strategy	FLowJo V10 software was used to analyze .fcs data and re-gating cells by FSC and SSC to sort approximately 10^4 cells, analyze cell's Cy5 fluorescence intensity by channel 4.Cell apoptosis assays in PI and Annexin V Alexa Fluor647 stained and Quad gate for channel 3 and 4.

- [x] Tick this box to confirm that a figure exemplifying the gating strategy is provided in the Supplementary Information.
